# Role of *N*-terminal protein formylation in central metabolic processes in *Staphylococcus aureus*

**DOI:** 10.1186/1471-2180-13-7

**Published:** 2013-01-16

**Authors:** Diana Mader, Manuel Liebeke, Volker Winstel, Karen Methling, Martina Leibig, Friedrich Götz, Michael Lalk, Andreas Peschel

**Affiliations:** 1 Interfaculty Institute of Microbiology and Infection Medicine Tübingen, Cellular and Molecular Microbiology, University of Tübingen, Elfriede-Aulhorn-Straße 6, 72076, Tübingen, Germany; 2 Institute of Pharmacy, University of Greifswald, Friedrich-Ludwig-Jahn Straße 17, 17487, Greifswald, Germany; 3 Interfaculty Institute of Microbiology and Infection Medicine, Microbial Genetics, University Tübingen, Waldhäuser Strasse 70/8, Tübingen, 72076, Germany; 4 Current address: Biomolecular Medicine, Department of Surgery and Cancer, Faculty of Medicine, Imperial College London, London, SW7 2AZ, UK

**Keywords:** Protein formylation, Bacterial metabolism, *Staphylococcus*, pyruvate dehydrogenase

## Abstract

**Background:**

Bacterial protein biosynthesis usually depends on a formylated methionyl start tRNA but *Staphylococcus aureus* is viable in the absence of Fmt, the tRNA^Met^ formyl transferase. *fmt* mutants exhibit reduced growth rates indicating that the function of certain proteins depends on formylated N-termini but it has remained unclear, which cellular processes are abrogated by the lack of formylation.

**Results:**

In order to elucidate how global metabolic processes are affected by the absence of formylated proteins the exometabolome of an *S. aureus fmt* mutant was compared with that of the parental strain and the transcription of corresponding enzymes was analyzed to identify possible regulatory changes. The mutant consumed glucose and other carbon sources slower than the wild type. While the turnover of several metabolites remained unaltered *fmt* inactivation led to increases pyruvate release and, concomitantly, reduced pyruvate dehydrogenase activity. In parallel, the release of the pyruvate-derived metabolites lactate, acetoin, and alanine was reduced. The anaerobic degradation of arginine was also reduced in the *fmt* mutant compared to the wild-type strain. Moreover, the lack of formylated proteins caused increased susceptibility to the antibiotics trimethoprim and sulamethoxazole suggesting that folic acid-dependant pathways were perturbed in the mutant.

**Conclusions:**

These data indicate that formylated proteins are crucial for specific bacterial metabolic processes and they may help to understand why it has remained important during bacterial evolution to initiate protein biosynthesis with a formylated tRNA^Met^.

## Background

The start of protein biosynthesis with a formylated methionine represents a distinct bacterial feature that is absent in eukaryotes [1,2]. The ubiquitous presence in all bacterial branches including mitochondria and chloroplast indicates a very important role of this trait in central bacterial cellular processes but it has remained unclear, which bacterial proteins depend on N-formylation for correct function. Nevertheless, it has become clear that formylation of the initiator tRNA is not essential for viability in some bacteria including *Staphylococcus aureus* where inactivation of the formyl transferase Fmt only leads to reduced growth and fitness [3,4].

The production of formylated proteins is potentially detrimental for bacterial pathogens because formylated peptides are sensed by mammalian innate immune systems leading to altered host defense and inflammation [5]. The human formyl peptide receptor FPR1 expressed on neutrophils and other leukocytes elicits neutrophil chemotaxis and activation upon ligand binding [6]. We have recently shown that formylated peptides represent crucial bacterial pathogen-associated molecular patterns [7] and that increased production of formylated peptides by inhibition of the deformylation reaction can increase proinflammatory reactions [8]. Of note, *S. aureus* secretes CHIPS, a potent inhibitor of FPR1 to interfere with immune activation [9].

The methionyl group of the bacterial start tRNA is modified by Fmt using formyl tetrahydrofolic acid (formyl-THF) as the formyl group donor [10]. Formyl-THF can be regenerated by different pathways among them the utilization of free formate in *S. aureus* produced by fermentation under anaerobic conditions [11]. The formyl group is removed from many proteins upon translation by polypeptide deformylase and this reaction is essential because the function of many proteins appears to depend on deformylated N-termini [12]. Accordingly, deformylase represents an attractive target for antibiotics [13]. Deformylase modifies only proteins with certain sequence motifs next to formyl-methionine while those with unfavorable N-terminal sequences remain unmodified [14]. The severe growth defect of Fmt mutants indicates that many bacterial proteins are fully functional only if the N-terminal formyl group is retained but it has remained unclear, which proteins these are. A recent proteomic study has shown by 2D gel electropheresis that the majority of proteins in *Bacillus subtilis* are deformylated but that a substantial number of proteins retain the formyl group [15].

In an attempt to elucidate how the absence of formylated proteins impacts on the metabolic capacities of bacteria the exometabolomes, abilities to catabolize specific nutrients, and susceptibilities to inhibitors of the folic acid metabolisms of *S. aureus* wild type and *fmt* mutant strains were compared. The results indicate that formylated proteins are required for distinct metabolic pathways including the anaerobic degradation of arginine via the arginine deiminase pathway and the oxidation of pyruvate. Moreover, the *fmt* mutant was more susceptible to trimethoprim and sulfamethoxazole indicating that the folic acid metabolism was perturbed in the mutant.

## Results

### Reduced growth of the *S. aureus* Δ*fmt* mutant in the presence of oxygen

**Figure 1:**
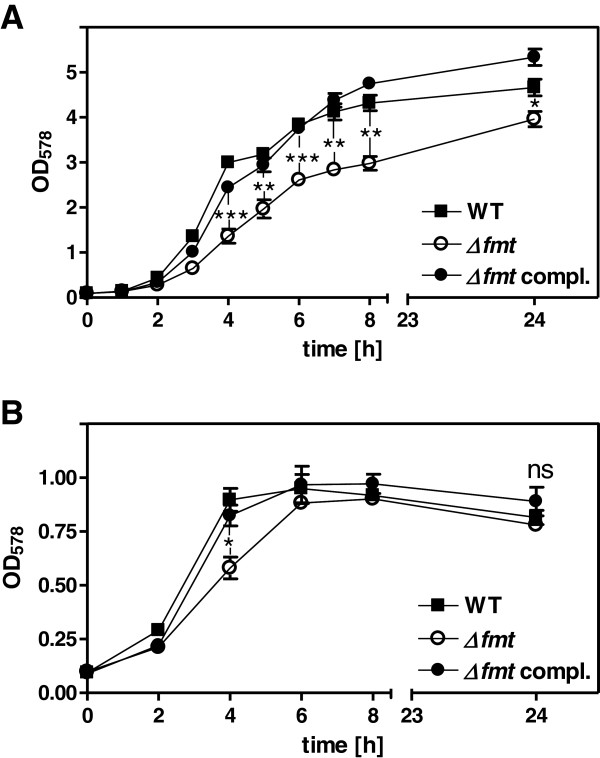
**Growth of Δ*fmt *mutant, wild type, and complemented Δ*fmt *mutant in BM under (A) aerated and (B) anaerobic conditions.** Data represent means ± SEM of three independent experiments. Significances of wild type vs. *Δfmt:* *P < 0.05; **P < 0.005; ***P < 0.001; ns not significant; as calculated with the two-tailed Student’s *t*-test.

The *fmt* gene is not essential for viability but its inactivation compromises growth in several bacterial species [3,4,16]. In order to analyze under which conditions *fmt* inactivation affects growth of *S. aureus* the multiplication of RN4220 wild type, *fmt* mutant (Δ*fmt),* and complemented mutant was monitored under aerated and non-aerated growth conditions. In the presence of oxygen Δ*fmt* exhibited a significantly reduced growth rate compared to wild type and complemented mutant and reached slightly lower densities after 24 h of growth (Figure 1A). Under anaerobic conditions growth of all three strains was similar and the mutant exhibited significantly lower densities only at the 4 h time point (Figure 1B).

It can be assumed that the growth defect of Δ*fmt* results largely from inactivity of proteins whose function may depend on N-terminal formylation. In order to elucidate how the absence of protein formylation may impact on global gene expression microarray analysis was performed with the wild-type and mutant strains. This experiment proved the absence of the *fmt* gene and showed that polypeptide deformylase, which has no substrates in the mutant is downregulated in Δ*fmt* (Table 1). In addition, genes from several metabolic pathways were downregulated in Δ*fmt* indicating that the absence of formylated proteins has pleiotrophic effects on transcription, which results probably either from dysfunctional regulatory proteins or from regulatory feedback events in metabolic pathways depending on formylated enzymes (see below).

**Table 1:** **Genes involved in metabolic processes differentially regulated by *fmt *deletion in *S. aureus *RN4220 under (A) aerobic or (B) anaerobic growth conditions**

**Gene ID**^**a,b**^	**Name**^**b**^	**Gene product**^b^	**x-fold change**
**A**			
**Reduced expression in Δ*fmt *compared to wild type:**			
Amino acid metabolism			
01452	*ald*	alanine dehydrogenase	103.1
00008	*hutH*	histidine ammonia-lyase	67.1
01451	*ilvA*	threonine dehydratase	39.8
00899	*argG*	argininosuccinate synthase	22.5
00435	*gltB*	glutamate synthase, large subunit, putative	21.8
02468	*alsS*	acetolactate synthase	14.1
00558		acetyl-CoA acetyltransferase, putative	12.2
01497	*ansA*	L-asparaginase, putative	7.6
01450		amino acid permease*	6.4
00081		HPCH-HPAI aldolase family protein*	4.6
02287	*leuC*	3-isopropylmalate dehydratase, large subunit	4.4
02574		NAD-NADP octopine-nopaline dehydrogenase family protein*	3.8
01450		amino acid permease*	3.2
02281	*ilvD*	dihydroxy-acid dehydratase	3.2
02839		L-serine dehydratase, iron-sulfur-dependent, alpha subunit	2.9
00510	*cysE*	serine acetyltransferase, putative	2.8
00147		acetylglutamate kinase, putative	2.5
02563	*ureF*	urease accessory protein, putative	2.3
02723		glycerate kinase, putative	2.2
Protein biosynthesis			
01183	*fmt*	methionyl tRNA formyltransferase	585.8
01182	def2*	polypeptide deformylase (def2*)	6.3
01839	*tyrS*	tyrosyl-tRNA synthetase	2.8
00324		ribosomal-protein-serine acetyltransferase, putative	2.4
01738	*hisS*	histidyl-tRNA synthetase	2.4
Folic acid metabolism			
01183	*fmt*	methionyl tRNA formyltransferase	585.8
02374		aminobenzoyl-glutamate utilization protein B, putative	4.5
02610	*hutG*	formiminoglutamase	3.4
Fermentation			
00188	*pflA*	formate acetyltransferase activating enzyme	604.5
02830	*ddh*	D-lactate dehydrogenase, putative	263.6
00187	*pflB*	formate acetyltransferase (pyruvate-formate-lyase)	99.0
00608	*adh1*	alcohol dehydrogenase I, putative	74.0
00113	*adhE*	alcohol dehydrogenase, iron-containing	40.8
02467	*budA2*	alpha-acetolactate decarboxylase	2.6
02875		L-lactate dehydrogenase, putative	2.3
Purine metabolism			
02553		inosine-uridine preferring nucleoside hydrolase*	3.3
00211		inosine-uridine preferring nucleoside hydrolase*	3.3
Lipid biosynthesis			
01278	*glpD*	aerobic glycerol-3-phosphate dehydrogenase	14.7
Transport systems			
00748		iron compound ABC transporter, ATP-binding protein, putative*	15.0
03019		ABC transporter, ATP-binding protein, putative	7.2
01991		ABC transporter, permease protein, putative	7.1
00155		PTS system, glucose-specific component	5.0
00424		ABC transporter, permease protein, putative	3.9
02154		ABC transporter, ATP-binding protein, putative	2.6
00844		ABC transporter, substrate-binding protein*	2.2
00215		PTS system component, putative	2.1
Urea metabolism			
00899	*argG*	argininosuccinate synthase	22.5
02563	*ureF*	urease accessory protein, putative	2.3
energy production and conversion/electrone transfer			
00412	*ndhF*	NADH dehydrogenase subunit 5, putative	359.0
00302		NADH-dependent flavin oxidoreductase, Oye family*	5.2
**Higher expression in Δ*fmt *compared to wild type:**			
Amino acid metabolism			
02971	*aur*	aureolysin, putative	3.4
**B**			
**Gene ID**^**a,b**^	**Name**^**b**^	**Gene product**^**b**^	**x-fold change**
**Reduced expression in Δ*fmt *compared to wild type:**			
Amino acid metabolism			
00836	*gcvH*	glycine cleavage system H protein	2.4
00151		branched-chain amino acid transport system II carrier protein	2.4
01452	*ald*	alanine dehydrogenase	2.3
01450		amino acid permease*	2.1
00510	*cysE*	serine acetyltransferase, putative	2.1
01451	*ilvA*	threonine dehydratase	2.1
Protein biosynthesis			
01183	*fmt*	methionyl-tRNA formyltransferase	158.3
01182	def2*	polypeptide deformylase (def2*)	4
01788	*thrS*	threonyl-tRNA synthetase	3.7
00009	*serS*	seryl-tRNA synthetase	2.4
01839	*tyrS*	tyrosyl-tRNA synthetase	2.3
01159	*ilsS*	isoleucyl-tRNA synthetase	2.1
Folic acid metabolism			
01183	*fmt*	methionyl-tRNA formyltransferase	158.3
00836	*gcvH*	glycine cleavage system H protein	2.4
Lipid biosynthesis			
01310		cardiolipin synthetase, putative	2.8
Fermentation			
02830	*ddh*	D-lactate dehydrogenase, putative	9.8
00206		L-lactate dehydrogenase	2.3
00113	*adhE*	alcohol dehydrogenase, iron-containing	2
**Increased expression in Δ*fmt *compared to wild type:**			
Amino acid metabolism			
02840		L-serine dehydratase, iron-sulfur-dependent, beta subunit	4.3
Protein biosynthesis			
01725		tRNA methyl transferase, putative	2.1
Purine metabolism			
01012	*purQ*	phosphoribosylformylglycinamidine synthase I	4.2
01014	*purF*	amidophosphoribosyltransferase	3.6
00372	*xprT*	xanthine phosphoribosyltransferase	3.2
Purine metabolism (*continued*)			
00375	*guaA*	GMP synthase, putative	2
Lipid biosynthesis			
01260	*pgsA*	CDP-diacylglycerol--glycerol-3-phosphate 3-phosphatidyltransferase	2.1
03006		lipase	2.7
Carbohydrate metabolism			
01794	*gap*	glyceraldehyde-3-phosphate dehydrogenase, type I	6.3
00239		ribokinase, putative	2.1
Riboflavin metabolism			
01886		riboflavin synthase, beta subunit	25
01888		riboflavin synthase, alpha subunit	5.7
01889	*ribD*	riboflavin biosynthesis protein RibD	4.5

* defined for *S. aureus* COL;

^a^ SAOUHSC gene ID for *S. aureus* NCTC8325.

^b^ Gene IDs, names and products are based on AureusDB (*http://aureusdb.biologie.uni-greifswald.de*) and NCBI (*http://www.ncbi.nlm.nih.gov/)* annotation.

### Absence of formylated proteins results in abrogated pyruvate metabolism

**Figure 2:**
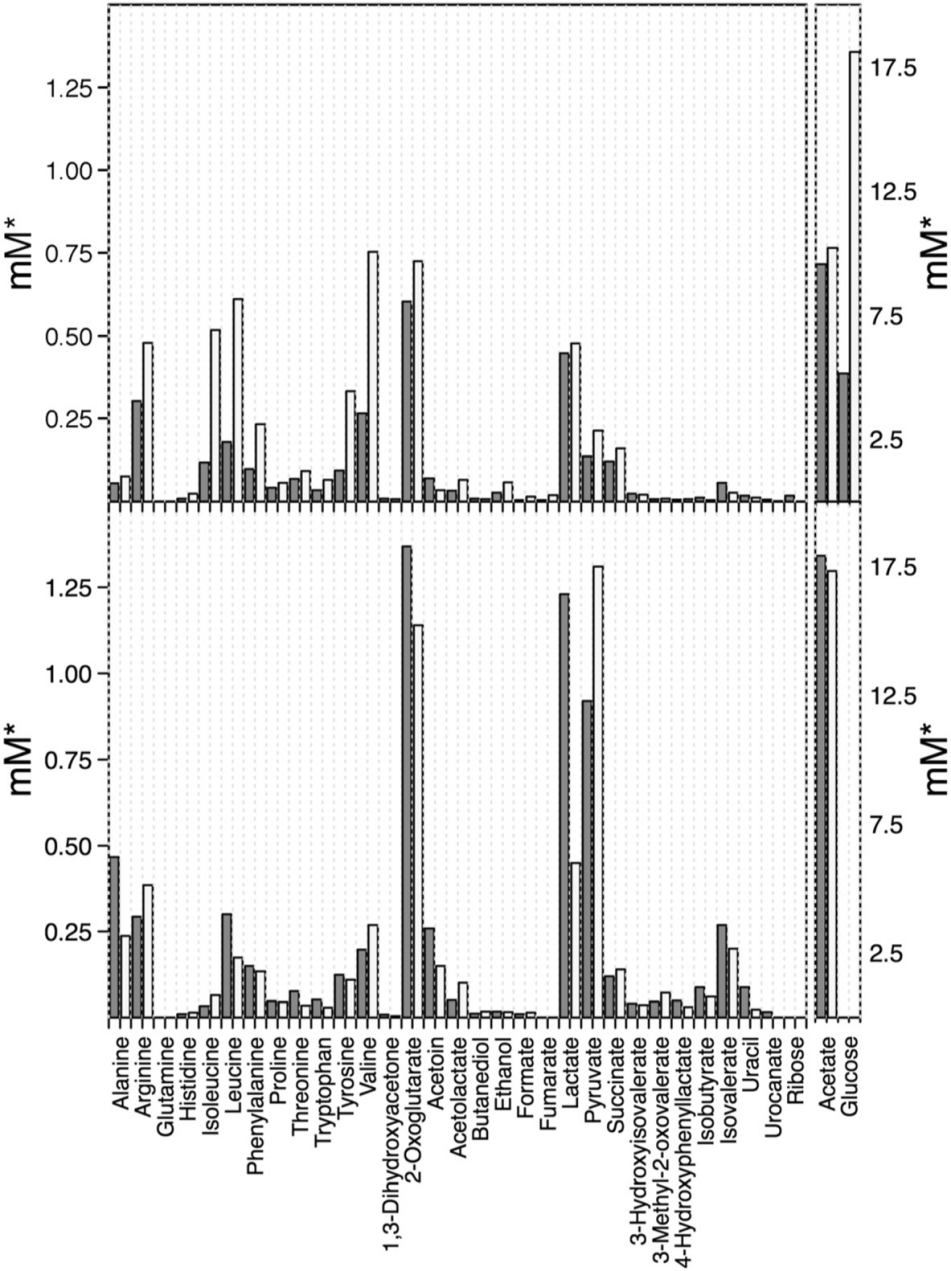
**Exometabolome analysis of *S. aureus *wild type (gray bars) and Δ*fmt *mutant (white bars) grown to late exponential (top) and stationary (bottom) growth phase.** *, concentrations relative to measured A_578_ values at a given time point.

In order to analyze how *fmt* inactivation affects global metabolic processes the exometabolomes of wild-type and Δ*fmt* strains in aerated cultures were compared by ^1^H-NMR in the exponential and stationary growth phases. Δ*fmt* consumed glucose much less efficiently than the wild type during the exponential growth phase, which is in agreement with the slower multiplication of the mutant but glucose was completely spent by both strains in the stationary phase (Figure 2). In parallel, arginine, branched-chain amino acids, and the aromatic amino acids phenylalanine and tyrosine were consumed more slowly by Δ*fmt* compared to the wild type during exponential growth but these differences disappeared largely in the stationary phase.

Both strains accumulated acetate, the primary catabolic product of *S. aureus* in aerated cultures [17] at similar levels and there were also no major differences found for the citric acid cycle intermediates 2-oxoglutarate, succinate, and fumarate. These findings suggested that central catabolic pathways downstream of acetyl-CoA were not affected by the lack of formylation in Δ*fmt*.

Of note, Δ*fmt* released more of the central metabolic intermediate pyruvate to the growth medium than the wild type in the stationary phase suggesting that the metabolism of pyruvate was perturbed in the absence of protein formylation. Pyruvate and acetyl CoA-derived fermentation products including acetoin, butanediol, ethanol, and lactate were produced by both strains indicating that growth conditions were not fully aerobic (Figure 2). However, Δ*fmt* produced considerably lower amounts of acetoin and lactate than the wild type, in particular in the stationary phase, which was paralleled by reduced expression of acetolactate decarboxylase and of two lactate dehydrogenases that lead to acetoin and lactate generation, respectively, from pyruvate (Table 1, Figure 2). Both strains produced alanine, which is generated from pyruvate by alanine dehydrogenase Ald, in the stationary phase. However, Δ*fmt* produced much less alanine, which corresponded to strongly reduced *ald* transcription in the mutant. Transcription of the four subunits of the pyruvate dehydrogenase complex PdhABCD was unaltered indicating that this major pyruvate-oxidizing enzyme linking glycolysis with the citric acid cycle should be present at similar amounts in wild type and Δ*fmt*. However, when cytoplasmic PdhABCD activity was compared the mutant exhibited ca. 20% lower activity than the wild type and complemented mutant (108 mU/mg protein vs. 133 mU/mg and 124 mU/mg, respectively) suggesting that in addition to reduced fermentative pyruvate reduction a lower pyruvate oxidation rate may contribute to increased pyruvate accumulation in the mutant. In agreement with these findings Δ*fmt* was found to have a higher molecular NAD^+^/NADH ratio compared to the wild-type strain (37.5 vs. 22.0, respectively). This difference may be a reason for some of the observed differences in expression of genes such as lactate dehydrogenase or alanine dehydrogenase, whose transcription is controlled by the NAD^+^-sensing Rex repressor [18]*.*

Microarray analyses did not reveal differences in expression of major enzymes involved in glycolysis or degradation of those amino acids that were less efficiently consumed by the mutant (Table 1). Thus, the reduced consumption of glucose or amino acids may result either from perturbed pyruvate utilization or/and from reduced activity of one or several enzymes involved in catabolic pathways upstream of pyruvate. Several genes involved in amino acid biosynthesis, protein and folic acid metabolism, and several transport systems were dysregulated in Δ*fmt*, which may also contribute to the slower growth of the mutant. Transcription of a putative NADH dehydrogenase subunit (*ndhF*) was strongly repressed in Δ*fmt,* maybe as a result of the altered NAD^+^/NADH ratio. However, Δ*fmt* grew much better under aerated compared to non-aerated conditions (Figure 1) and it did not produce more ermentation products than the wild type (Figure 2) indicating that the respiratory capacity of the mutant remained largely intact. Δ*fmt* also released lower amounts of uracil than the wild-type (Figure 2) and this difference was reflected by reduced expression of uridine nucleoside hydrolase (Table 1A).

### Lack of arginine deiminase activity in Δ*fmt* mutant

**Figure 3:**
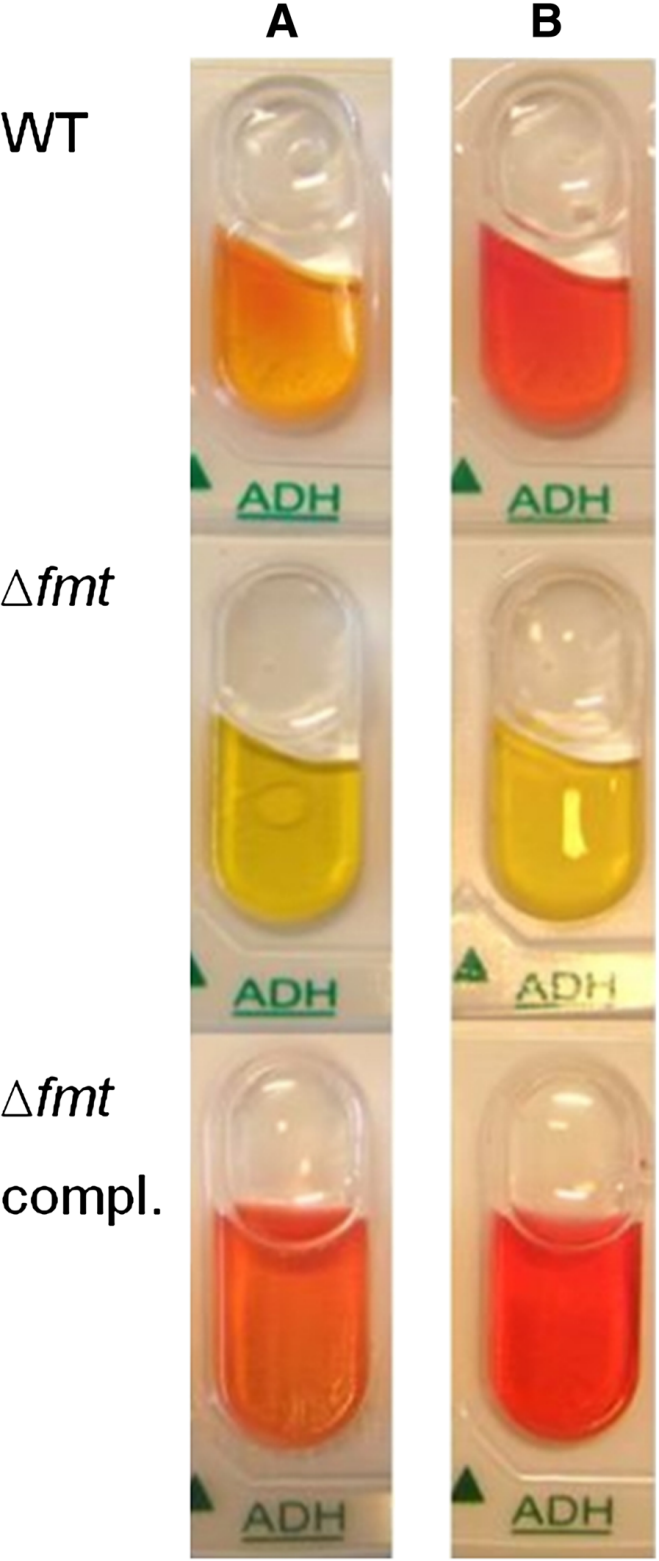
Δ***fmt *is not able to deiminate arginine.** ApiStaph tests (BioMérieux) were performed with the wild type, Δ*fmt* mutant, and complemented Δ*fmt* mutant and photographically evaluated after (**A**) 24 h and (**B**) 30 h incubation under anaerobic conditions.

Our metabolomics approach measured only those metabolites that appeared in culture supernatants. In order to monitor further metabolic activities the wild-type, *Δfmt* and complemented mutant strains were checked for the ability to catabolize different carbon and energy sources with an ApiStaph diagnostic test (BioMérieux). Only one out of 20 reactions revealed a different behavior of Δ*fmt* (Figure 3). No degradation of arginine via arginine deiminase (ADI) leading to the production of citrulline and ammonia was observed in Δ*fmt*. This reaction is the first step in the anaerobic catabolism of arginine, which serves as an ATP source by substrate level phosphorylation [19]. Of note, the enzymes of the ADI pathway were not altered in their expression, neither under aerobic nor anaerobic conditions (Table 1) suggesting that the absence of formylation may directly affect the activity of one or more ADI pathway enzymes.

### *fmt* inactivation leads to increased susceptibility to trimethoprim and sulfamethoxazole

**Figure 4:**
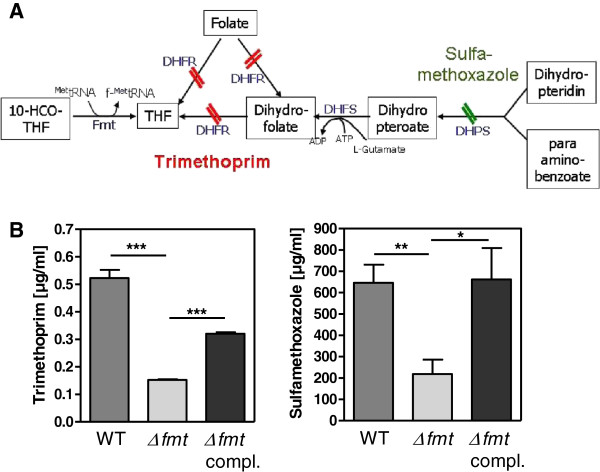
**Impact of Δ*fmt *mutation on the folic acid pathway. **(**A**) Consequences on folic acid metabolism of dihydrofolate reductase (DHFR) inhibition with trimethoprim and dihydropteroate synthetase (DHPS) inhibition with sulfamethoxazole. DHFS stands for dihydrofolate synthase. (**B**) MICs were determined for the indicated antibiotics. Data represent means ± SEM of at least three independent experiments. *P < 0.05; **P < 0.005; ***P < 0.001 as calculated by the two-tailed Student’s *t*-test.

In order to analyse if the block in formyl transfer from 10-CHO-THF to the initiator tRNA impacts on susceptibility to inhibitors of folic acid metabolic processes the abilities of trimethoprim and sulfamethoxazole, antagonists of dihydrofolate reductase and dihydropteroate synthetase, respectively [20], to inhibit *S. aureus* with or without functional Fmt were compared. In fact, the MICs of trimethoprim and sulfamethoxazole were 3.5-fold and 3-fold lower, respectively, in Δ*fmt* compared to the parental strain (Figure 4). Complementation with a plasmid-encoded copy of *fmt* led to partially or fully restored MICs indicating that the increased susceptibility of Δ*fmt* was in fact a result of lacking formylated proteins. No changes in expression of genes for key enzymes directly involved in the folic acid pathway were observed (Table 1) indicating that the proteins should be produced in equal amounts but may differ in activities leading to altered metabolic fluxes and corresponding differences in antagonist susceptibilities.

## Discussion

Our study demonstrates that the lack of start tRNA ormylation has pleiotropic consequences and affects the global *S. aureus* exometabolome and transcriptome in multiple ways. Protein N-termini are usually positively charged but formylated amino groups cannot be protonated any more, which can alter protein conformation and function substantially. We expected that protein dysfunction resulting from N-terminal charge alteration may affect cellular functions in multiple ways including e.g. by compromising the function of structural proteins, regulators, or enzymes leading to global cellular stress responses, altering regulatory networks, or perturbing metabolic pathways, respectively. It has remained unclear, which *S. aureus* proteins retain formyl groups upon translation and the activity of which of these may depend on formylation. Our approach set out to assess, which metabolic processes may be compromised in a *fmt* mutant and we found that many exometabolites were present at similar levels in the wild-type and Δ*fmt* strains while the catabolism of glucose and certain amino acids, the release of pyruvate or pyruvate-derived fermentation products, and the susceptibility to inhibitors of enzymes depending on folic acid derivatives was changed. Thus, protein formylation has distinct roles in certain metabolic pathways. The reduced catabolism of glucose and branched-chain and aromatic amino acids by Δ*fmt* was not reflected by changes in transcription of genes for corresponding enzymes suggesting that these changes did not result from perturbed gene regulation but from compromised abilities of *S. aureus* to degrade these nutrients. Likewise, the anaerobic degradation of arginine by the ADI pathway was abolished in Δ*fmt* while transcription of the corresponding genes appeared to be unchanged. It remains unclear, which of the many catabolic enzymes may be affected by the lack of N-terminal protein formylation. Moreover, we noted that transcription of some transport proteins of unknown function was reduced in Δ*fmt* and it cannot be ruled out that one or several of these may be required for amino acid uptake.

Extracellular accumulation of the central metabolic intermediate pyruvate was much more pronounced in Δ*fmt* than in the wild type, which was accompanied by reduced production of pyruvate-derived alanine and fermentation products acetoin and lactate. The production of fermentation products suggests that our cultivation conditions were not fully aerobic. The concomitantly reduced transcription of alanine dehydrogenase, acetolactate decarboxylase, and lactate dehydrogenases suggests that pyruvate accumulation may be a result of transcriptional repression of fermentative pathways in Δ*fmt* the reasons for which remain unknown and may result e.g. from altered activity of metabolic regulators such as the NAD^+^-sensing Rex [18]. However, the specific activity of the pyruvate-oxidizing PDHC was also reduced in the mutant, which is in accord with the increased NAD^+^/NADH ratio in the mutant and our recent finding that inhibition of *S. aureus* PDHC leads to accumulation of extracellular pyruvate [21]. Since transcription of the PDHC-encoding genes *pdhABCD* was unaltered in Δ*fmt* its reduced PDHC activity may indicate that one or several proteins of PdhABCD may require a formylated N-terminus for full activity.

Since inactivation of Fmt should lead to increased amounts of formyl THF and reduced amounts of free THF in Δ*fmt* we proposed that the mutant should have altered susceptibility to antibiotics that block the de novo synthesis of THF. In fact, Δ*fmt* was more susceptible to trimethoprim and sulfamethoxazole than the wild type, which indicates that the folic acid metabolism was perturbed by *fmt* inactivation and suggests that the availability of THF derivatives that are e.g. necessary for purine biosynthesis becomes growth-limiting at lower antibiotic concentrations as in the wild type.

## Conclusions

Our study shows that the lack of protein formylation does not abrogate all kinds of metabolic activities but has particular impacts in certain pathways. Elucidating, which specific enzymes or regulators may lose their activity by the lack of formylation remains a challenging aim. Our approach will be of importance for defining individual metabolic pathways depending on formylated proteins and it represents a basis for more detailed studies. Addressing these questions will not only be of importance for understanding a central bacterial process, it may also help to identify new antibiotic targets and further elucidate the importance of formylated peptides in innate immune recognition.

## Methods

### Bacterial strains and growth

*S. aureus* RN4220 and the previously described RN4220 *fmt* mutant [4] along with the plasmid-complemented mutant [7] were used in this study. In order to compare growth kinetics basic medium (BM) composed of 1% casein peptone, 0.5% yeast extract, 0.5% NaCl, 0.1% K_2_HPO_4_ × 3 H_2_0, and 0.1% glucose was inoculated with bacterial over-night cultures grown in tryptic soy broth (TSB; Fluka) at an OD_578_ of 0.08 and cultivated either with aeration (50 ml in notched 100 ml flasks on a shaker) or without (completely filled, sealed 15 ml tubes) at 37°C and OD_578_ was measured at several time points. Cultures of the complemented mutant were supplemented with 10 μg/ml chloramphenicol. To compare capacities to catabolize various substrates the various strains were used to inoculate ApiStaph tubes (BioMérieux), which were incubated and evaluated according to the manufacturers’ manual.

### Extracellular metabolome analysis by ^1^H-NMR

For quantification of extracellular metabolites TSB overnight cultures of RN4220 wild type and the Δ*fmt* mutant were used to inoculate 100 ml Iscove’s modified Dulbecco’s media (IMDM) without phenol red (Gibco) in notched 250 ml flasks at an OD_578_ of 0.1. The cultures were incubated on a shaker at 37°C. Samples were taken at 8 h and 24 h to determine the OD_578_ and obtain culture supernatants by centrifugation with subsequent filtration (0.22 μm pore size). Samples were prepared and analyzed by ^1^H-NMR as described recently [21,22]. Briefly, 400 μl of supernatants were mixed with 200 μl phosphate buffer (0.2 M; pH 7.0) and applied to a Bruker®Avance II 600 MHz spectrometer operating with TOPSPIN 2.0 (Bruker®Biospin). Metabolites were identified by comparison with pure reference compound spectra. Trimethylsilylpropionic acid d_4_ was used as internal standard. All spectra were processed in Chenomx NMR Suite 4.6 (Chenomx, Edmonton, AB, Canada) and selected metabolites were quantified by computer-assisted manual fitting of metabolite peaks.

### RNA isolation and microarray analyses

To compare the transcription profiles of the RN4220 wild type and Δ*fmt* mutant the strains were grown in BM (13 ml in notched 50 ml flasks) at 37°C to an OD_578_ 1.0 under aerobic conditions or to an OD_578_ 0.5 under anaerobic conditions (completely filled and sealed 15 ml tubes). Bacteria were harvested via centrifugation and immediately frozen at −80°C. Samples were then thawed on ice and resuspended with 1 ml Trizol (Invitrogen) to inhibit RNases and bacteria were disrupted with 0.5 ml glass bead suspension in a homogenizer. The supernatants of these lysates were mixed with 200 μl chloroform for 60 s and incubated for another three minutes to extract the RNA. After centrifugation (15 min; 12,000 × g; 4°C) the upper phase was collected and pipetted into 500 μl isopropanole. After 10 min at room temperature the samples were centrifuged for 30 min again to collect supernatants. Then 500 μl 70% ethanol was added and the samples were centrifuged at 4°C, 7,500 × g for 5 min. After removing the supernatants pellets were dried for about 1 h, dissloved in 500 μl sodium citrate buffer, and further purified with a Microcon filter cartridge (Millipore) and stored at −80°C. Each 10 μg of RNA from two biological replicates per condition and strain were applied to GeneChip microarrays (Affymetrix) and processed according to the manufacturer’s protocol. The biological replicates yielded highly reproducible expression profiles, which were deposited at the GEO data base (http://www.ncbi.nlm.nih.gov/geo/) with accession number GSE41713.

### Pyruvate dehydrogenase complex (PDHC) activity

*S. aureus* cells grown in BM to late exponential phase were resuspended in phosphate buffer (0.2 M, pH 7.4) and disrupted by a combined enzymatic (lysostaphin) and mechanical (glass bead mill) procedure in the presence of DNase I as described recently in detail [21]. Insoluble components were removed from the extracts by centrifugation (14,000 × *g* for 10 min at 4°C) and 4 × 500 μl of the resulting filter-sterilized lysate were subjected to ultracentrifugation for 1 h using a Beckman TLA-55 rotor at 50,000 rpm and 4°C to enrich PDHC. Reaction mixtures for determining PDHC activity contained 0.2 M phosphate buffer, 0.2 mM MgCl_2_, 0.01 mM CaCl_2_, 0.3 mM thiamine diphosphate, 0.12 mM coenzyme A (CoA), 2.0 mM ß-NAD^+^, 5.1 mM pyruvate, 0.1 mM 1-methoxy-5-methylphenazinium methyl sulphate (mPMS), and 0.4 mM iodonitrotetrazolium formazan in an assay volume of 1.5 ml. Enzymatic activity was measured spectrophotometrically at 500 nm and 20°C as described recently [21]. Units of activity were calculated using a molar absorption coefficient of 12.5 mM^-1^ cm^-1^.

### NAD^+^/NADH quantification

To measure alterations in the NAD^+^/NADH ratio between RN4220 wild type and Δ*fmt* the strains were grown in BM at 37°C to an OD_578_ of 1.0 under aerobic conditions. The NAD^+^/NADH Quantification Kit (BioVision) was used and processed according to the manufacturer’s protocol with some modifications. Briefly, 25 ml of the cultures were harvested by centrifugation and pellets were resuspended in 400 μl of NADH/NAD extraction buffer. Extracts were obtained by homogenizing the resuspended pellets with 0.5 ml glass bead suspension. After centrifugation the supernatants were filtered through 10 kDa molecular-weight cut-off filters (BioVision). Ratios were calculated as [total NAD minus NADH]/NADH.

### Minimal inhibitory concentration of antibiotics

To define differences in the susceptibility to trimethoprim and sulfamethoxazole (Sigma) over-night cultures of RN4220 wild type, Δ*fmt,* and the complemented mutant were used to inoculate 500 μl IMDM without phenol red (Gibco) to an OD_578_ of 0.1 in 24-well plates (Costar) containing serially diluted antibiotics in duplicates. After 18 hours incubation at 37°C under gentle agitation the densities were measured to determine minimal inhibitory concentrations.

## Competing interests

The authors declare to have no competing interests.

## Authors’ contributions

DM, MLi, VW, KM, ML performed the experiments; FG, MLa, AP conceived the study; AP wrote the manuscript. All authors read and approved the final manuscript.
